# Therapeutic effects of functional orthodontic appliances on cervical spine posture: a retrospective cephalometric study

**DOI:** 10.1186/1746-160X-10-7

**Published:** 2014-03-24

**Authors:** Maren Ohnmeiß, Gero Kinzinger, Julia Wesselbaum, Heike M Korbmacher-Steiner

**Affiliations:** 1 Private praxis, Bahnhofstr, 55/1, Leonberg 71229, Germany; 2 Department of Orthodontics, University Hospital Saarland, Kirrberger Str. 100, Homburg/Saar 66424, Germany; 3 Department of Orthodontics, University Hospital Giessen and Marburg, Georg-Voigt-Strasse 3, Marburg 35039, Germany

**Keywords:** Angle class II/1, Functional orthodontics, Craniovertebral junction, Cervical spine posture

## Abstract

**Introduction:**

Interactions between the cervical spine and the stomatognathic system have been discussed in literature. The present study was conducted to investigate whether, and to what extent, orthodontically induced mandibular advancement produces changes in cervical spine posture. Furthermore, possible appliance-specific effects should be distinguished.

**Material and methods:**

The cephalograms of 64 patients with skeletal class II were analysed before and after mandibular advancement. Linear and angular cephalometric parameters were identified to define the position of the atlanto-occipital and atlantoaxial joints. The total example was divided into two subgroups (comprising 32 individuals each) according to the employed appliance: activator versus bite-jump appliance (BJA). Student's t-test and analysis of covariance were used for statistical analysis.

**Results:**

Overall, a significant straightening of the cervical spine was observed during the treatment. This conclusion is based on changes of Chamberlain (p = 0.0055), CVT (p = 0.0003), OPT (p < 0.0001), Redlund-Johnell/Petersson (p < 0.0001), McGregor-mC2 (p = 0.0333) and AT-FH (p = 0.0445). Improvements in occipitoatlantal dislocation were also observed in the total sample. Appliance-specific changes were found in the activator subgroup for a number of linear parameters (Chamberlain, McGregor, CVT, OPT, Redlund-Johnell/Petersson). In contrast, only two linear parameters (OPT and Powers ratio) revealed statistically significant changes in the BJA subgroup.

**Conclusions:**

During skeletal class II treatment the position of upper cervical spine changes. In the activator subgroup the observed effects were more pronounced than those in the BJA subgroup. Further studies including a control group comprised with non-treated class II patients are needed to assess whether these effects may be caused directly by the appliances irrespective of growth.

## Introduction

There is agreement in literature that pathological orthopedic findings are highly prevalent among individuals with orthodontic anomalies [1-5]. These observations have been explained by anatomical, phylogenetic and functional interactions between the masticatory system and the upper cervical spine. Numerous authors have devoted attention to the relationship between occlusal anomalies and spinal disorders or deformities. Duyzings [6] reported an association between the postural inclination of the cervical spine and the position of the mandible. Prager [7] demonstrated that the prevalence of malpositioned teeth and jaw anomalies were significantly increased in patients with spinal deformities. Functional interactions of a predominantly morphologic and neuromuscular nature have been suspected to influence the entire system of cranial, cervical, dorsal and sacral structures in such a way that any disturbance of one segment would affect the entire system [8].

Angle class II.1 has been shown to be associated with an atlas inferior position, a habitual lack of an upright head posture and a lordosis of the cervical spine. In contrast, Angle class III has been demonstrated to involve an atlas superior position and a kyphosis of the cervical spine [9]. Although many studies have revealed orthodontic and orthopedic interactions, only a few interdisciplinary treatment approaches have been recommended so far: Early orthodontic correction of unilateral crossbite should be regarded as mandatory in patients with scoliosis or torticollis in order to minimize the facial asymmetry related to the orthopedic problem and to stabilize head position [10,11].

Animal experiments have shown that changes of the occlusal height and jaw position led to changes of the upper cervical spine and evoked reactions of the motor and autonomic nervous system [12]. Fink et al. [8] also demonstrated that mandibular advancement led to changes within the craniocervical system and within the region of lumbar, pelvic and hip structures.

Angle class II.1 is the most prevalent anomaly. During growth, orthodontic appliances can produce the orthodontically desired skeletal changes. While the skeletal and profile-changing effects of functional orthodontics in class II patients are widely documented in literature [13,14]. None of these reports have specifically addressed changes possibly occurring at the craniocervical level. Therefore, cephalograms, which had been obtained in the context of functional orthodontic treatment of skeletal class II patients, were analysed in terms of changes in the craniocervical level. Furthermore, it should be evaluated whether the effects were appliance specific.

## Materials and methods

### Patients

The study was conducted according to the Helsinki Declaration. The study design was approved by the Ethic committee of the RWTH Aachen university (reference number AZ 171/08). In this retrospective study only patients with distal occlusion ranging from 0.5 to 1 premolar width, a protruded upper incisor inclination and an ANB angle > 4° were included. Cases involving gnathic deviation of the mandible and/ or transversal discrepancy were excluded. The successful use of either an activator or a BJA for skeletal treatment was required. A total of 64 patients (35 female and 28 male) with a mean age of 11 years and 2 months met these criteria. The mean skeletal treatment duration was 12 months and 7 days. Appliances were selected according to therapeutic requirements, using an activator for the correction of distal occlusion only and a BJA whenever additional indications for single-tooth movement and/or transversal development of the maxilla were needed. The following null hypothesis was proposed: the skeletal correction of class II evokes changes of the articulations of the craniovertebral junction.

### Cephalometry

Two cephalograms of each patient were available: the first was taken at the beginning; the second after skeletal treatment had been successfully completed. A standardized technique of image acquisition had been used throughout, with the teeth in habitual occlusion and the central beam passing through the porus acusticus externus perpendicular to the film plane. All cephalograms were evaluated by Ricketts analysis (Table 1) in order to assess orthodontic effects and growth.

**Table 1:** Angles and distances indicating skeletal change in the sagittal and vertical planes

**Parameters**	**Active group**	**p-values**	**BJA group**	**p-values**	**All patients**	**p-values**
SNA (°)	-0.76 ± 1.71	0.0155*	-0.81 ± 2.66	0.1347	-0.78 ± 2.10	0.0045*
SNB (°)	0.24 ± 2.27	0.5489	0.09 ± 231	0.8465	0.2 ± 2.20	0.4733
ANB (°)	-0.99 ± 1.30	0.0001**	-0.93 ± 1.16	0.0004**	-0.99 ± 1.23	< 0.0001**
BjØrk sum^a^ (°)	0.72 ± 4.74	0.3904	-1.69 ± 9.46	0.3691	-0.26 ± 7.02	0.7709
Gonion angle^b^ (°)	-0.12 ± 2.31	0.7764	-0.80 ± 4.16	0.3344	-0.41 ± 3.15	0.3095
S-Go (mm)	2.78 ± 4.01	0.0004*	3.03 ± 3.49	0.0002**	3.00 ± 3.71	< 0.0001**
N-Me (mm)	4.81 ± 3.48	<0.0001**	4.39 ± 5.04	0.0002**	4.69 ± 4.12	< 0.0001**
SN/ML (°)	0.85 ± 3.72	0.1991	0.38 ± 3.38	0.5746	0.61 ± 3.44	0.1642
PE/ML (°)	0.78 ± 2.55	0.0890	-0.22 ± 3.45	0.7446	0.30 ± 2.95	0.4178

Data are given as mean differences (± SD) between posttreatment and baseline values.

Asterisks (* or **) indicate significant (p < 0.05) or highly significant (p < 0.001) differences.

^a^N-S-Ar + S-Ar-Go + Ar-Go-Me; ^b^Ar-Go-Me.

### Parameters

**Figure 1:**
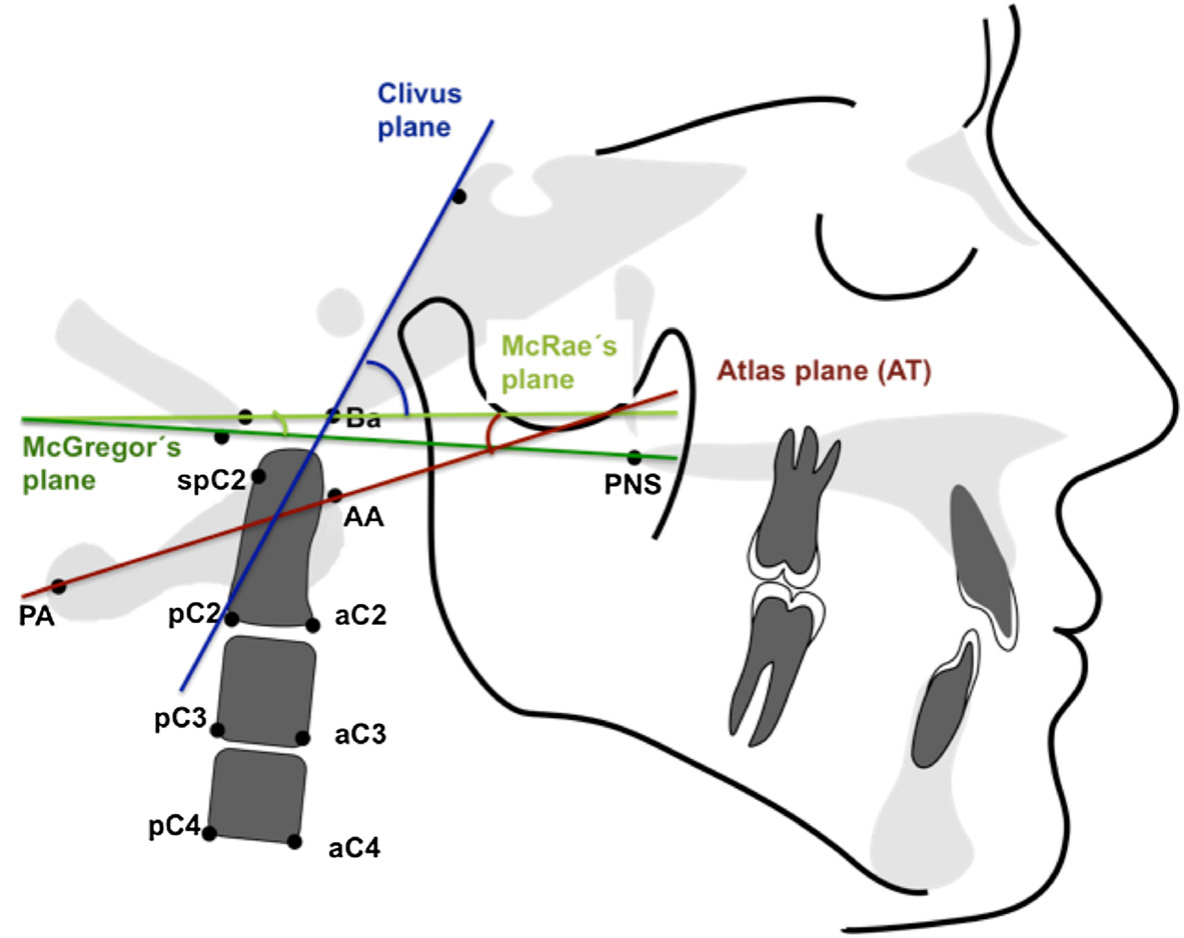
Angular and linear parameters of head posture and position.

**Figure 2:**
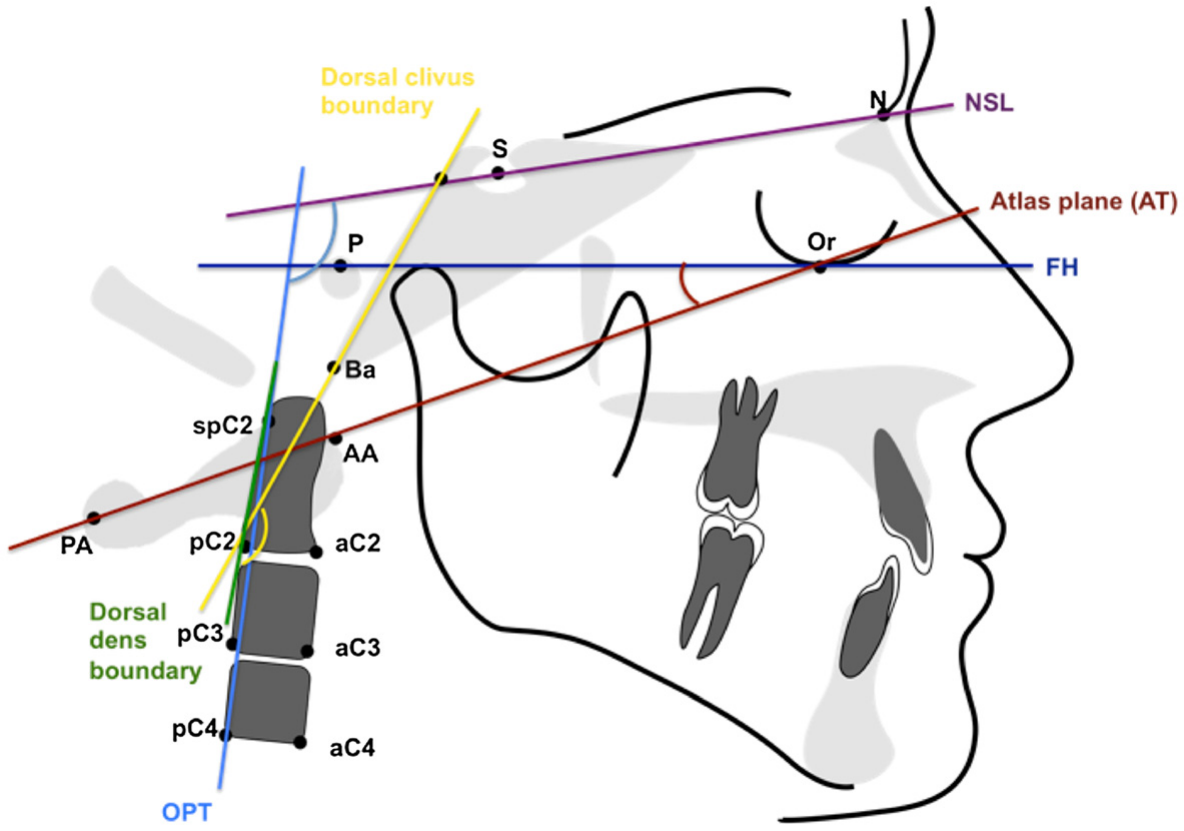
Angular and linear parameters of cervical spine straightening.

**Figure 3:**
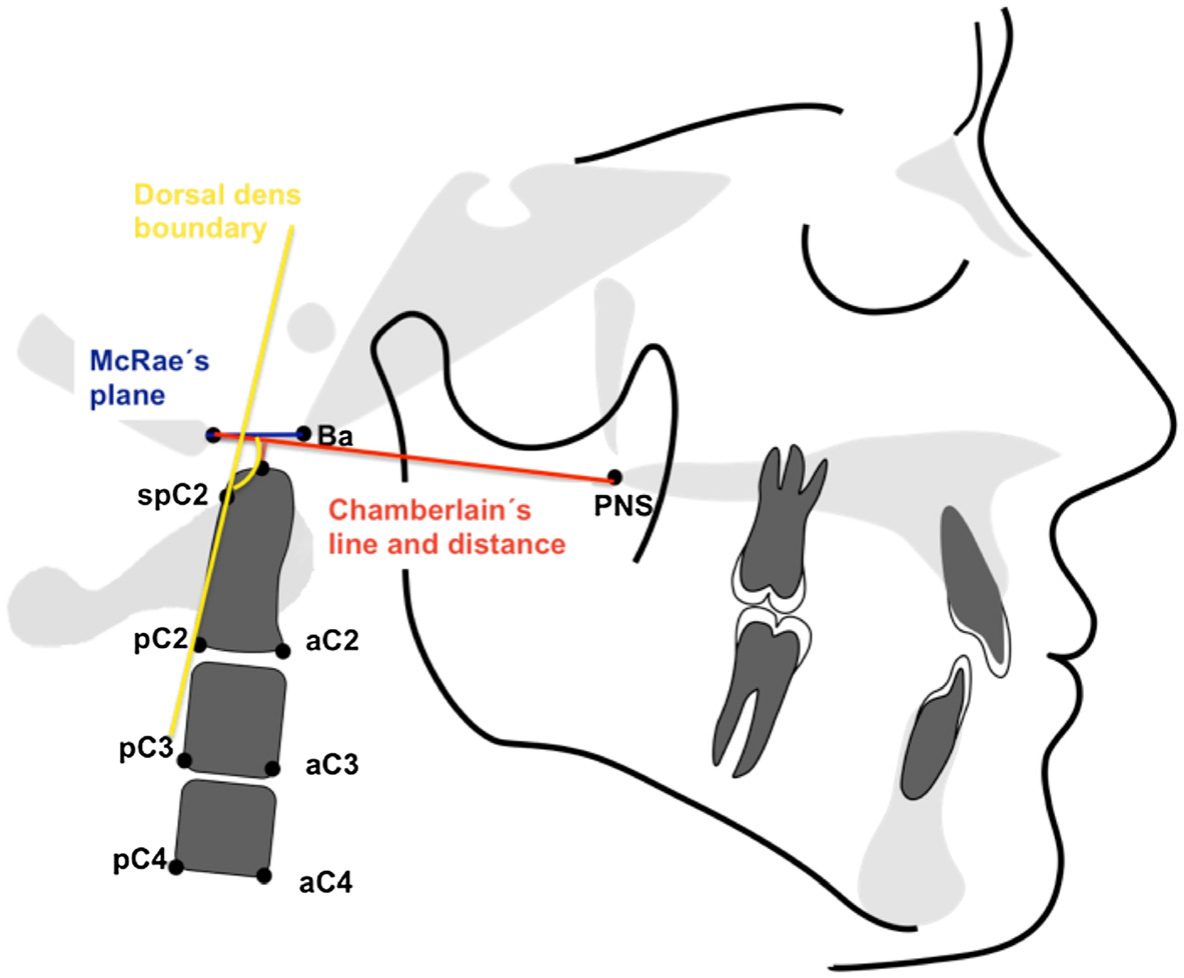
Angular and linear parameters of basilar impression.

**Figure 4:**
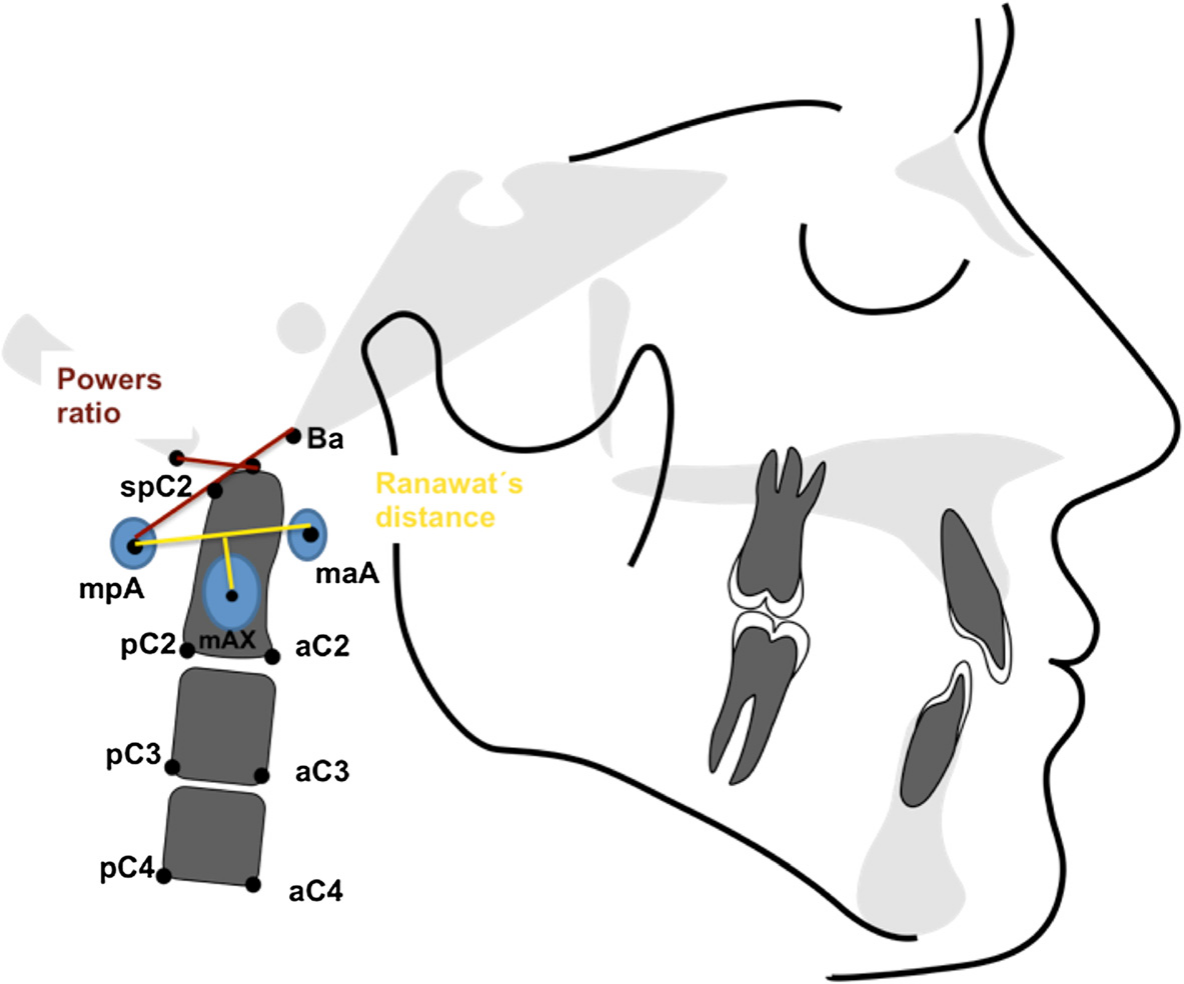
Angular and linear parameters of occipitoatlantal dislocation 1.

**Figure 5:**
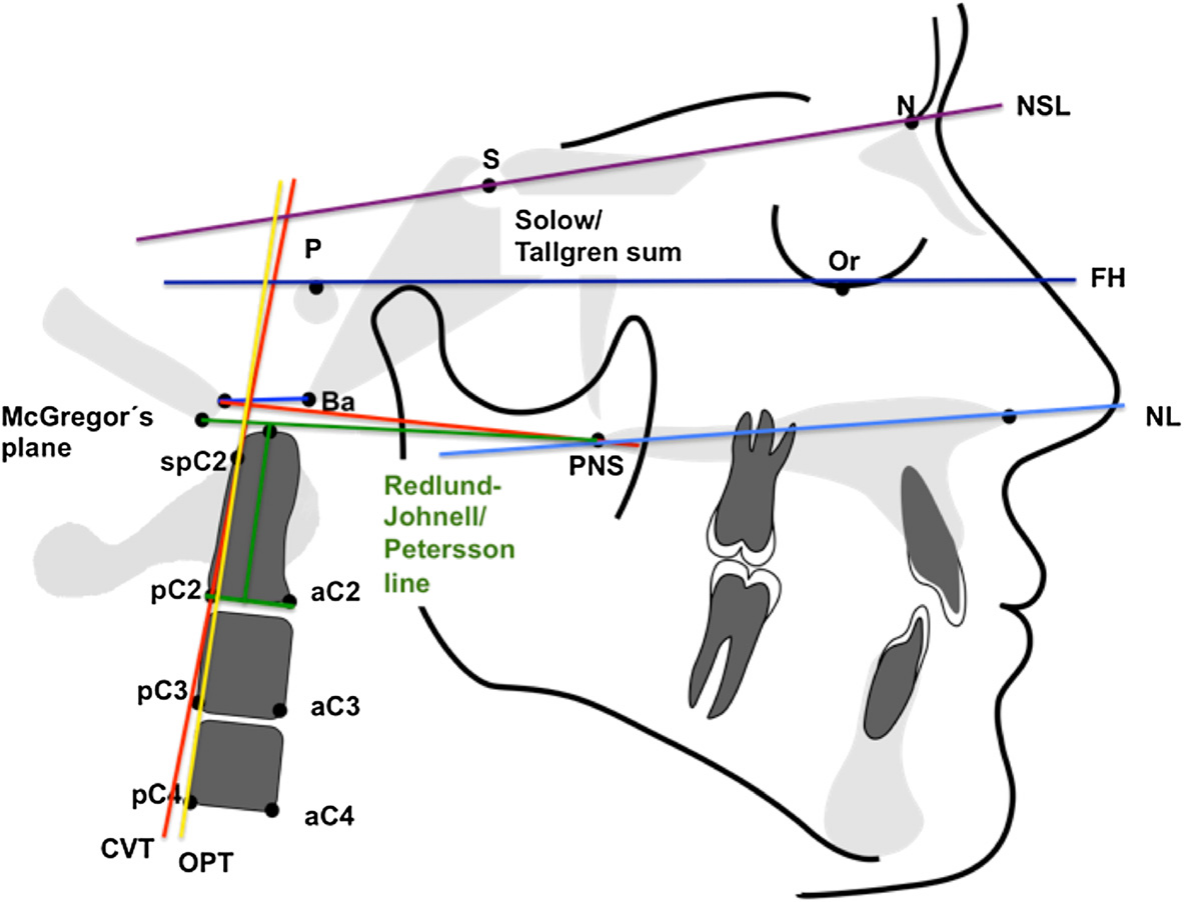
Angular and linear parameters of occipitoatlantal dislocation 2.

Table 2 summarizes the atlantoaxial linear and angular parameters used for orthopedic analysis. Head posture and position (Figure 1) were analyzed based on angular measurements of various planes (McGregor, atlas, clivus) relative to McRae’s plane. Straightening of the cervical spine (Figure 2) was determined based on the inclination of the atlas toward the Frankfort plane, the clivus-dens angle, and the angle formed by the OPT line against the anterior cranial base. Basilar impression (Figure 3) refers to cranial displacement of the cervical vertebrae, causing the dens to project itself at the level of the foramen magnum; its presence was verified by measuring the angle of McRae’s line to the dorsal dens boundary and the distance from Chamberlain’s line to the dens. Occipitoatlantal dislocation (Figures 4 and 5) refers to a shifted position of the atlas relative to the cranial base; it was assessed by the Powers ratio and Ranawat’s line, also using the approach by Redlund-Johnell and Petersson of measuring the distance from McGregor’s plane to the center of the inferior endplate of the second cervical vertebra, and the complementary technique by Solow and Tallgren of calculating the sum of four specific angles relative to the Frankfort plane.

**Table 2:** Orthopedic parameters, including landmarks and definitions

**Atlas inclination (modified)**	**Angle from atlas plane to Frankfort horizontal plane.**
**Atlas plane (AT)**	**Line drawn through the most anterior and most posterior sites of the atlas.**
**Chamberlain’s distance**	**Distance from dens tip to Chamberlain’s line.**
**Chamberlain’s line (palato-occipital line)**	**Line drawn from posterior edge of hard palate to posterior edge of foramen magnum.**
**Clivus-dens angle**	**Angle between dorsal ends of clivus and dens axis.**
**Dorsal clivus boundary**	**Dorsal end of clivus**
**Dorsal dens boundary**	**Dorsal end of dens axis**
**McGregor’s line (palato-suboccipital line)**	**Line from posterior edge of hard palate to most inferior point of squama occipitalis.**
**McRae’s line (foramen magnum line)**	**Line between anterior and posterior edges of foramen magnum.**
**OPT line**	**Line drawn against the anterior cranial base to define the craniocervical angle.**
**Powers ratio**	**Ratio between distances (i) opisthion to dens tip and (ii) Ba to projection center of arcus posterior atlantis.**
**Ranawat’s line**	**Distance between a line connecting the projection centers of the anterior/posterior arches of the atlas and the center of the shadow of the axis vertebra.**
**Redlund-Johnell/Petersson line**	**Distance between McGregor’s line and center of inferior endplate of second cervical vertebra (mC2)**
**Solow/Tallgren sum (modified)**	**Sum of angles formed by linear parameters OPT, CVT (line through spC2 und pC2), NL, NSL and ML to the Frankfort horizontal plane.**

### Measurements

After scanning the cephalograms, the linear and angular measurements were performed using diagnostic software (Fr win; Computer Konkret AG, Falkenstein, Germany). To ensure comparability among the different cephalograms, the enlargement factor of each cephalogram was individually determined and multiplied for all linear measurements. Double contours (related to the radiographic technology used) were averaged.

### Statistical analysis

Spreadsheet (Microsoft Excel 2007) and statistics (SAS Version 9.1; SAS Institute Inc., Cary, NC) software was used to analyze data, calculating arithmetic means and standard deviations for all cephalometric parameters. Analysis of covariance was used to evaluate whether any of the findings were linked to the use of the orthodontic appliance. Student’s t-test was performed in order to identify any significance between the first and second measurement. The resultant p-values were considered statistically significant at < 0.05. Normal distribution of the various parameters had been verified beforehand, and the t-test was performed in duplicate – once summarily for all data and once separately for each orthodontic appliance. Analysis of covariance was employed to find out whether the use of a specific orthodontic appliance was linked to any of the values obtained from the posttreatment cephalograms, using the corresponding baseline values as covariable. Multivariate regression yielded no statistically significant difference between both groups with regard to skeletal age, baseline skeletal findings, duration of treatment, or effect of treatment. Hence both groups were comparable (see Table 1). All cephalograms of the same patients were analyzed twice by the same investigator and checked for efficiency.

### Systematic error

Dahlberg’s combined systematic error [13] was calculated using the formula MF = √(∑d^2^/2n), where “d” is the difference between two measurements and “n” the number of measurements performed in duplicate. Twenty cephalograms were arbitrarily selected and reanalyzed 3 months after first analysis. The mean values thus obtained were 0.6° and 0.41 mm.

## Results

**Figure 6:**
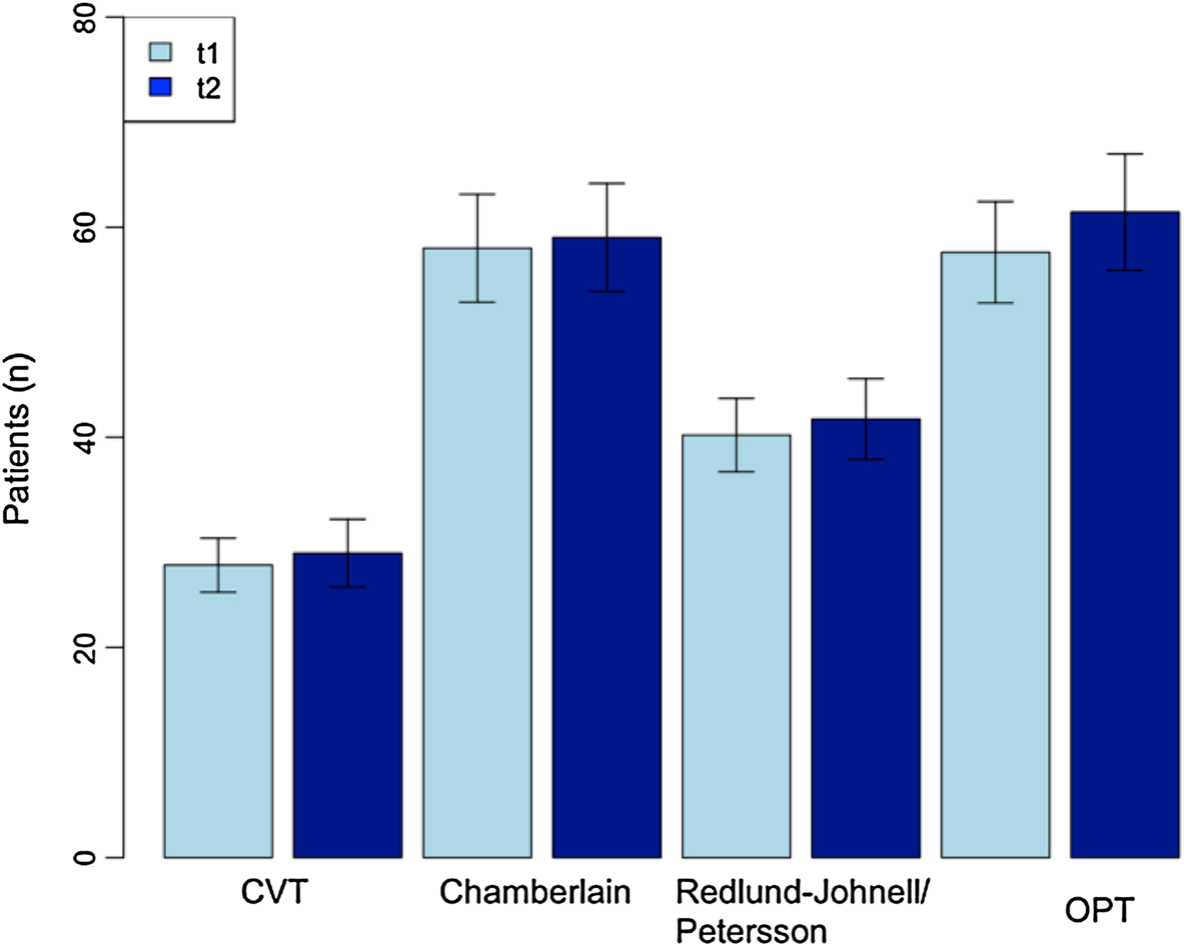
Bar chart of significant (*) and highly significant (**) differences between atlantoaxial linear measurements before (t1) and after (t2) skeletal treatment.

Mean values and standard deviations were calculated for the various (angular and linear) orthopedic values derived from the cephalograms in both appliance-specific patient groups. Figure 6 illustrates significant changes obtained for a number of atlantoaxial linear parameters based on the total sample of patients, including significant changes for Chamberlain by 0.93 mm ± 2.58 mm (p = 0.0055) and highly significant changes for CVT (p = 0.0003), OPT (p < 0.0001) and Redlund-Johnell/Petersson (p < 0.0001). Atlantoaxial distances increased in both treatment approaches. Based on the total sample of patients, the atlantoaxial angles revealed no major changes, while occipitoatlantal dislocation and basilar impression were falling short of statistical significance (Table 3).

**Table 3:** Atlantoaxial angular measurements (summary)

**Angular parameters (°)**	**All patients**	**p-values**
Solow/Tallgren sum (modified)		
OPT-FH	0.75 ± 7.85	0.4535
CVT-FH	-0.67 ± 7.99	0.5102
NL-FH	-0.12 ± 2.91	0.7433
NSL-FH	0.43 ± 2.73	0.2136
Clivus-dens angle	0.23 ± 7.92	0.8207
McGregor-CVT	0.11 ± 7.56	0.9075
McGregor-CVT	-6.67 ± 43.34	0.2267
McGregor-AT	0.33 ± 5.92	0.7227
AT-CVT	-0.41 ± 5.45	0.6328
(Ba-C)-McRae	0.67 ± 61.96	0.9324
(Ba-C)-AT	0.51 ± 4.59	0.4747
McRae-AT	16.05 ± 74.93	0.1726
Craniocervical angle (NSL-OPT)	-0.53 ± 8.18	0.6054
Atlas inclination (AT-FH)	-0.09 ± 4.27	0.8987

Data are given as mean differences (± SD) between posttreatment and baseline values.

Taken the different appliances into account, significant changes of atlantoaxial linear parameters were seen within the activator group (Table 4). The activator group revealed significant changes for Chamberlain (p = 0.0034) and McGregor (p = 0.0283) and highly significant changes for CVT (p = 0.0006), OPT (p < 0.0001) and Redlund-Johnell/Petersson (p < 0.0001). In the BJA group, only Powers ratio as the only linear parameter underwent significant changes; it decreased by 3.18 ± 6.1 mm (p = 0.0477). In the activator group, no significant changes were seen in any of the atlantoaxial angular parameters. Significant changes in the BJA group were only seen for the NL-FH angle (p = 0.0064), thus being confined to one isolated parameter that was evaluated as part of the Solow/Tallgren sum of angles (Table 5).

**Table 4:** Atlantoaxial linear measurements (appliance-specific evaluation)

**Linear parameter (mm)**	**Active group**	**p-values**	**BJA group**	**p-values**
McRae (Ba-opisthion)	0.25 ± 1.46	0.3291	0.38 ± 2.37	0.4199
Chamberlain (opisthion-PNS)	1.28 ± 2.33	0.0034*	0.69 ± 2.95	0.2394
McGregor (PNS-SO)	1.12 ± 2.80	0.0283*	-0.29 ± 4.00	0.7127
CVT	1.78 ± 2.66	0.0006**	0.33 ± 2.05	0.4174
OPT	4.74 ± 3.73	< 0.0001**	2.69 ± 4.01	0.0021*
AT	-0.02 ± 1.92	0.9589	-0.67 ± 2.72	0.3543
Ranawat (mpA-maA-maX)	0.35 ± 1.39	0.2005	-0.08 ± 1.11	0.7639
Powers ratio (SO-D/Ba-apA)	1.74 ± 6.31	0.1564	-3.18 ± 6.11	0.0477*
Chamberlain’s distance	-0.13 ± 2.35	0.7466	-0.21 ± 2.15	0.6200
Redlund-Johnell/Petersson (McGregor-mC2)	2.07 ± 2.29	< 0.0001**	0.85 ± 2.40	0.0835

Data are given as mean differences (± SD) between posttreatment and baseline values.

p-values refer to intragroup comparisons.

Asterisks (* or **) indicate significant (p < 0.05) or highly significant (p < 0.001) differences.

**Table 5:** Atlantoaxial angular measurements (appliance-specific evaluation)

**Angular parameter**	**Active group**	**p-values**	**BJA group**	**p-values**
Solow/Tallgren sum (modified)				
OPT-FH	0.98 ± 8.15	0.4867	0.20 ± 7.76	0.8945
CVT-FH	0.17 ± 9.58	0.9181	-2.14 ± 5.62	0.0634
NL-FH	-0.16 ± 1.71	0.6000	-0.00 ± 4.13	0.0064*
NSL-FH	0.24 ± 2.13	0.5174	0.6 ± 3.51	0.3917
Clivus-dens angle	0.00 ± 9.55	0.9986	0.89 ± 5.55	0.4222
McGregor-CVT	0.17 ± 7.68	0.8998	-0.01 ± 7.49	0.9938
McGregor-CVT	-1.58 ± 5.65	0.1193	-14.90 ± 66.98	0.2623
McGregor-AT	0.14 ± 5.82	0.9052	0.47 ± 6.57	0.7845
AT-CVT	-0.65 ± 5.37	0.5562	-0.45 ± 5.98	0.7733
(Ba-C)-McRae	9.93 ± 60.47	0.3525	-11.95 ± 67.48	0.3753
(Ba-C)-AT	0.69 ± 5.35	0.5259	-0.02 ± 3.46	0.9825
McRae-AT	12.42 ± 70.11	0.3847	23.05 ± 89.05	0.3332
Craniocervical angle (NSL-OPT)	0.35 ± 9.29	0.8308	-2.13 ± 6.71	0.1174
Atlas inclination (AT-FH)	-0.84 ± 4.66	0.3875	1.2 ± 3.68	0.2267

Data are given as mean differences (± SD) between posttreatment and baseline values.

p-values refer to intragroup comparisons.

Asterisks indicate significant (p < 0.05) or highly significant (p < 0.001) differences.

## Discussion

The present study demonstrates that cervical spine posture changes during treatment with functional orthodontic appliances. This is in accordance with Sonnesen et al. [15], who found craniocervical angles to be enlarged in the presence of dysgnathia and hyperlordosis and to be reduced by orthodontic treatment.

Interactions between the masticatory system and the cervical spine have been increasingly discussed over the years. Back in 1926, Schwarz [16] observed an association between head posture and jaw position. Head posture has been discussed to influence the mode of breathing during sleep and to have effects on craniofacial growth. Gresham and Smithells [9] furnished radiographic evidence that children habitually lacking an upright head posture reveal an Angle class II, a long-face syndrome, and enhanced lordosis of the cervical spine. This latter observation was confirmed by Balters [17]. Radiographic findings by Treuenfels and Torklus [18] suggested interactions between atlas position, dysgnathia and head posture. Hirschfelder and Hirschfelder [2] on the other hand did not confirm an Angle class characteristic atlas position. Mertensmeier and Diedrich [19] observed hyperlordosis of the cervical spine in over 40% of patients with class I or class II anomalies. Fink et al. [8] also demonstrated that occlusal changes have functional implications both within the craniocervical system and in the body area comprising the lumbar, pelvic and hip structures.

The cervical spine changes observed in the present study must be discussed as causally related to orthodontic treatment and/or produced by growth. In general, the straightening of the cervical spine is orthopedically desirable and consistent with physiological straightening during growth observed in Angle class II patients. In agreement with other studies prior orthodontic correction, the children revealed occipitoatlantal dislocation, basilar impression, hyperlordosis of the cervical spine, and retroflexion of the head [3,20,21]. A persistence of those findings led to atlas displacement, descendence of the hyoid, reducement of the pharyngeal size, persistence of mouth breathing, and further retrusion of the mandible [22]. Therefore, the straightening is an important therapeutic aspect.

These considerations raise the question to what extend orthodontic appliances are capable of straightening the cervical spine. Based on the total sample of patients, we were able to document a significant change of orthopedic parameters. Significant changes were more pronounced in the activator group. Our explanation for this finding is offered by the “Norwegian” activator system introduced by Andresen in 1935. The therapeutic effect of the activator is explained by the stimulation of masticatory muscles, lips and tongue, thereby transmitting functional stimuli to surrounding hard structures such as tooth, bone, and cervical spine [23,24].

It appears that the mandibular advancement had an impact on the straightening of the cervical spine. From an orthopedic view, such straightening is consistent with physiological growth and therefore would seem to be desirable. As part of the observed changes were due to physiological growth, their causation can be attributed to a combination of orthodontic treatment and ongoing growth independent of the conducted treatment.

From the methodic point of view the used linear orthopedic parameters (Chamberlain, OPT, CVT, Redlund-Johnell/Petersson) must be critically discussed: Values obtained for Chamberlain’s distance are potentially distorted by difficulties in marking the posterior edge of the foramen magnum and the double contours of the hard palate [25]. McGregor’s plane, which we used for Redlund-Johnell/Petersson analysis, offers the most reliable information of the linear parameters used [20,26]. In addition to yielding well-reproducible markings, the McGregor’s plane represents a stable reference plane not undergoing any growth-related changes [27]. Values within the normal range and without significant changes were obtained for Ranawat’s line, suggesting that this parameter also remains stable during physiological growth. All orthopedic reference values used in this study are gender-specific and based on adults only [28,29]. Since the patients in this study still grow, the cephalometric findings are bound to reflect combined effects of growth as well as treatment. In order to identify the net effect of treatment, a control group is needed in order to assess the growth effects involved. Since a historical control group with all relevant data has not been available in literature, the effects reported in this communication should be strictly regarded as gross effects.

## Conclusions

Numerous studies have demonstrated correlations between orthopedic and orthodontic findings, also with regard to specific anomalies being associated with characteristic spinal postures. The null hypothesis of our study was sustained. Quantitative evidence was furnished that the dens moved closer to the spheno-occipital complex and that the dens axis and atlas were verticalized during skeletal advancement of the mandible thus compensating for the characteristic finding of cervical spine hyperlordosis in class II patients. There was a tendency for these effects to be more pronounced in the activator group than in the BJA group. Our finding of cervical spine changes during orthodontic treatment highlights the usefulness of interdisciplinary collaboration especially in patients with orthopaedic abnormalities.

## Competing interests

The authors declare that they have no competing interests.

## Authors’ contributions

HKS carried out the study design, initiated the study and draft the manuscript. JW carried out the cephalometric analysis and participated in the data collection GK participated in the idea for this study, data collection, treated the patients . MO participated in the design of the study, performed the coordination of the study, helped to perform the cephalometric analysis and to draft the manuscript. All authors read and approved the final manuscript.
